# Impact of food waste addition in energy efficient municipal wastewater treatment by aerobic granular sludge process

**DOI:** 10.1007/s11356-024-32997-5

**Published:** 2024-04-04

**Authors:** Busra Cicekalan, Nastaran Rahimzadeh Berenji, Muhammed Furkan Aras, Huseyin Guven, Ismail Koyuncu, Mustafa Evren Ersahin, Hale Ozgun

**Affiliations:** 1 Civil Engineering Faculty, Department of Environmental Engineering, Istanbul Technical University, Maslak, Istanbul, 34469 Turkey; 2 National Research Center On Membrane Technologies, Istanbul Technical University, Maslak, Istanbul, 34469 Turkey

**Keywords:** Aerobic granular sludge, Biochemical methane potential, Co-digestion, Co-treatment, Food waste, Municipal wastewater

## Abstract

Recently, one of the main purposes of wastewater treatment plants is to achieve a neutral or positive energy balance while meeting the discharge criteria. Aerobic granular sludge (AGS) technology is a promising technology that has low energy and footprint requirements as well as high treatment performance. The effect of co-treatment of municipal wastewater and food waste (FW) on the treatment performance, granule morphology, and settling behavior of the granules was investigated in the study. A biochemical methane potential (BMP) test was also performed to assess the methane potential of mono- and co-digestion of the excess sludge from the AGS process. The addition of FW into wastewater enhanced the nutrient treatment efficiency in the AGS process. BMP of the excess sludge from the AGS process fed with the mixture of wastewater and FW (195 ± 17 mL CH_4_/g VS) was slightly higher than BMP of excess sludge from the AGS process fed with solely wastewater (173 ± 16 mL CH_4_/g VS). The highest methane yield was observed for co-digestion of excess sludge from the AGS process and FW, which was 312 ± 8 mL CH_4_/g VS. Integration of FW as a co-substrate in the AGS process would potentially enhance energy recovery and the quality of effluent in municipal wastewater treatment.

**Graphical abstract:**

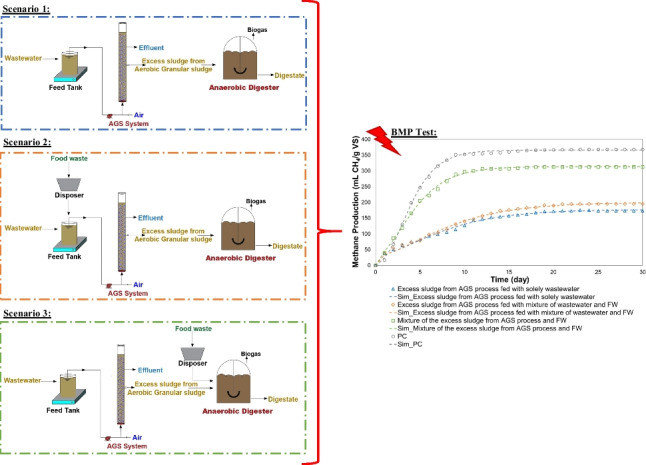

**Supplementary Information:**

The online version contains supplementary material available at 10.1007/s11356-024-32997-5.

## Introduction

With increasing urbanization and industrialization, the assimilation capacity of the environment has been decreasing. Therefore, the treatment of wastewater has become a critical task for public and environmental sanitation. Biological treatment is the most frequent method in wastewater treatment to remove pollutants discharged into the aquatic environment. Biological nutrient removal (BNR) systems are widely used for nitrogen and phosphorus removal from wastewater by appropriate microorganisms under different operational conditions. Among conventional BNR processes, the A^2^O process has a wide range of uses in wastewater treatment. The A^2^O process has anaerobic-anoxic-aerobic compartments operating at various redox potentials and a settling tank for the separation of liquid–solid. The main drawbacks of the A^2^O process are the high footprint and energy requirement (Nancharaiah et al. 2019). Thus, the need for novel treatment technologies is increasing to eliminate these bottlenecks for efficient wastewater treatment. Research on biological wastewater treatment is currently directed toward resource recovery (Franca et al. 2018; Guven et al. 2023). Aerobic granular sludge (AGS) technology, an innovative technology that permits simultaneous carbon and nutrient removal, has been increasingly used for municipal wastewater treatment (de Bruin et al. 2004; de Kreuk et al. 2007; Winkler et al. 2015; Kosar et al. 2022a; Cicekalan et al. 2023). Compared to conventional activated sludge processes using flocculent sludge, the AGS process provides better settling ability and higher biomass retention having the ability to withstand high-strength wastewater and shock load (Zheng et al. 2005).

Aerobic granulation has been fundamentally acquired in sequencing batch reactors (SBR) and is strongly associated with operation conditions, which should be favorable for microorganisms to form aggregates and/or granular particles (Liu and Tay 2004). SBRs are operated in a cyclic mode that includes a series of phases such as anaerobic feeding, aeration, settling, idling, and discharge. Microorganisms are exposed to high substrate concentration in the feeding phase by means of discontinuous operation in SBRs, and hence, it allows the substrate to penetrate the inner layers of the granules. It was indicated that substrate cannot permeate into deeper layers in reactors that are continuously fed (Beun et al. 2002). To ensure nitrogen removal, the concentration of dissolved oxygen (DO) in the aeration phase is controlled so that the inner layers of granules are not sustained to a high level of DO. Such an air feeding regime promotes the growth of nitrifiers and denitrifiers in the outer and inner layers of granules, respectively (de Kreuk et al. 2005). Polyphosphate-accumulating organisms (PAOs) in the core of granules have become prominent in the AGS reactor fed under anaerobic conditions. Hence, the AGS process can provide enhanced biological phosphorus removal (EBPR) (Coma et al. 2012).

The most preferred approach for enhancing the energy self-sufficiency of wastewater treatment plants (WWTPs) is the anaerobic digestion of excess sludge (Ersahin 2018). There are several applications in order to enhance methane production from excess sludge (Silvestre et al. 2015; Guven et al. 2019; Abdelrahman et al. 2021). For example, anaerobic co-digestion can be described as an instantaneous digestion of two or more substrates. Anaerobic co-digestion provides benefits such as enhanced nutrient balance, the growth of microbial synergy, increasing the load of biodegradable organic matter, and improvement of methane production with high digestion rates (de Castro et al. 2020). The organic fraction of municipal solid waste (OFMSW) that involves a high amount of biodegradable matter is one of the most frequently applied co-substrate for co-digestion applications (Bolzonella et al. 2006). Food waste (FW) is an easily biodegradable substrate for anaerobic digestion with its high content of carbohydrates, lipids, protein, and moderate moisture. Co-digestion of FW and sewage sludge is an attractive approach for increasing methane yield (Mehariya et al. 2018). FW from households may be diverted to WWTPs via typical solid waste collection vehicles. Kitchen grinders, installed in households, can be considered an option for conveying organic solid waste into WWTPs (Mattsson et al. 2014). Hence, co-treatment of wastewater and FW was proposed as an alternative waste management concept to lessen the negative impacts of waste transportation on the environment as well as providing benefits at WWTPs through anaerobic digestion (Guven et al. 2019).

Several studies have been focused on the impact of co-treatment of municipal wastewater and FW on treatment performance and energy recovery in the last decades (Sankai et al. 1997; Bolzonella et al. 2003; Monino et al. 2016, 2017; Barrios-Hernández et al. 2020). A pilot-scale anaerobic membrane bioreactor (AnMBR) was operated for the co-treatment of municipal wastewater and FW (Monino et al. 2017). They found that increasing the influent COD caused a considerable enhancement in methane production. The effects of the co-treatment of synthetic fecal sludge and wastewater in an AGS system were investigated by Barrios-Hernández et al. (2020). It was indicated that the addition of fecal sludge in the AGS reactor decreased the settleability of granular sludge. OFMSW and FW are potential co-substrates for anaerobic digestion. Cabbai et al. (2013) found an improvement of 16% and 48% in methane yield when co-digesting sewage sludge with OFMSW and FW, respectively. In the study of Iqbal et al. (2022), the effect of FW integration with biological wastewater treatment was investigated by the life cycle assessment analysis method in terms of wastewater treatment performance and energy balance. Two scenarios were studied in that study: co-treatment of FW with wastewater + anaerobic digestion and anaerobic co-digestion of FW with sewage sludge. The FW addition enhanced the total methane production by ~ 73% and ~ 130% in the co-treatment of FW with wastewater + anaerobic digestion scenario and co-digestion of FW with sewage sludge scenario, respectively. The addition of FW did not have a negative impact on the operation of WWTPs in terms of treatment capacity and meeting the discharge criteria (Iqbal et al. 2022).

There are several studies in which FW was used as a co-substrate (Cabbai et al. 2013; Guven et al. 2019; Montecchio et al. 2019; Zan and Hao 2020). Although there are few studies available on the effect of co-treatment with several substrates in the AGS process in the literature, there is no study conducted on the investigation of FW as a co-substrate in the AGS process. The purpose of this study was to comprehensively explore the viability of the co-management of FW with municipal wastewater and excess sludge from the AGS process. The impact of co-treatment of municipal wastewater with FW on the morphology of granules and treatment performance of the AGS process was investigated. Besides, the biochemical methane potential (BMP) test was performed to determine the methane yields of the digestion of the excess sludge from the AGS system and co-digestion of the excess sludge from the AGS system with FW. The mass balance based on COD was performed to investigate the effect of the FW addition on methane production.

## Material and methods

### Experimental procedure

**Fig. 1:**
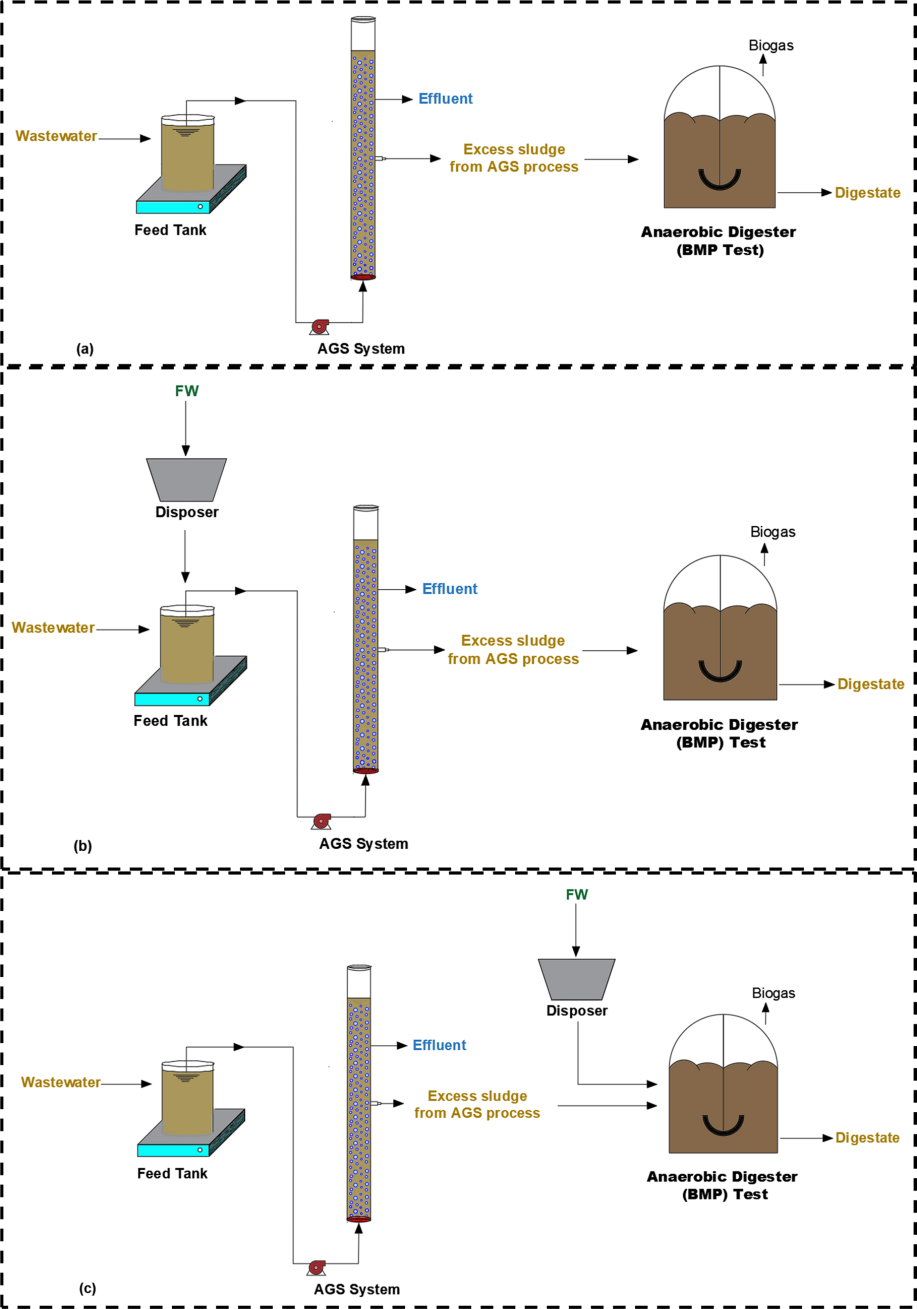
System configuration for each scenario: **a** Scenario 1, **b** Scenario 2, and **c** Scenario 3

To investigate the effect of co-treatment of wastewater and FW, two stages were used in this study. At stage 1, raw wastewater from a full-scale municipal WWTP was fed to a laboratory-scale AGS reactor, whereas a mixture of wastewater and FW was fed to the AGS reactor at stage 2. Afterward, to have a better understanding of the digestibility of the excess sludge from the AGS process, BMP tests were conducted with three different scenarios (Fig. 1):

- Scenario 1: The excess sludge (taken at stage 1) from the AGS process fed with solely wastewater was used for the BMP test.
- Scenario 2: The excess sludge (taken at stage 2) from the AGS process fed with the mixture of wastewater and FW was used for the BMP test.
- Scenario 3: Mixture of the excess sludge from the AGS process (taken at stage 1) and FW was used for the BMP test.

### Seed sludge characteristics

The laboratory-scale AGS system was seeded with the inoculum obtained from another laboratory-scale AGS system. The inoculum characteristics was indicated in Table 1. The ratio between volatile suspended solids (VSS) and total suspended solids (TSS) was 0.73 ± 0.09 with an average TSS concentration of 7200 ± 680 mg/L in the inoculum. The median particle size (d_50_) of the inoculum, and average granule diameter were 74.4 ± 0.6 µm, and 0.68 ± 0.25 mm, respectively.

**Table 1:** Seed sludge characteristics

Parameters	Unit	Value (average ± standard deviation (SD))
TSS	mg/L	7200 ± 680
VSS	mg/L	5160 ± 160
COD	mg/L	6870 ± 19
Soluble COD (sCOD)	mg/L	174 ± 2
Sludge volume index_5_ (SVI_5_)	mL/g	92 ± 16
Sludge volume index_10_ (SVI_10_)	mL/g	81 ± 2
Sludge volume index_30_ (SVI_30_)	mL/g	64 ± 3
SVI_5_/SVI_30_	mL/g	0.72 ± 0.10
SVI_10_/SVI_30_	mL/g	0.80 ± 0.05
Normalized capillary suction time (CST)	sec/g TSS/L	1.17 ± 0.03
pH	-	7.6 ± 0.1
d_50_	µm	74.4 ± 0.6
Mean granule size	mm	0.68 ± 0.25

### Substrate characteristics

The laboratory-scale AGS system was operated under two stages in the study. At stage 1, the AGS reactor was fed with raw wastewater, whereas the AGS reactor was fed with the mixture of wastewater and FW at stage 2. A commercial kitchen FW grinder (InSinkErator Model 45, UK) was used to obtain pulped FW. Pulped FW, composed of vegetable and fruit leftovers as well as bakery products on a roughly equal weight basis, was introduced to the AGS reactor. The FW was collected from a nearby cafeteria, grocery store, and bakery. It should be noted that the pulped FW did not include any fat oil grease. The wastewater and FW were sieved through a 2-mm mesh screen for removal of the coarse particles, and they were kept at 4 °C till use. Characterizations of the substrates are given in Table 2. At each stage, the AGS reactor was operated at the similar organic loading rate (OLR), 0.97 ± 0.03 kg COD/m^3^∙day and 0.96 ± 0.02 kg COD/m^3^∙day at stage 1 and stage 2, respectively. At stage 2, the relative contribution (as a percentage) of the wastewater and FW to OLR was 60% and 40%, respectively. The addition of FW led to an increase in total suspended solids (TSS) and organic matter in the influent. Calcium and magnesium concentrations at stage 1 were 116 ± 11 mg/L and 126 ± 36 mg/L, respectively, while they were 130 ± 8 mg/L and 140 ± 24 mg/L at Stage 2, respectively.

**Table 2:** Characterization of the substrates

Parameter	Unit	Stage 1	Stage 2		
		Wastewater (average ± SD)	Wastewater (average ± SD)	FW (average ± SD)	Mixture (wastewater + FW) (average ± SD)
TSS	mg/L	244 ± 11	253 ± 11	19,570 ± 625	346 ± 5
VSS	mg/L	193 ± 12	180 ± 7	18,728 ± 352	278 ± 4
VSS/TSS	-	0.81 ± 0.03	0.71 ± 0.03	0.96 ± 0.02	0.80 ± 0.02
COD	mg/L	361 ± 12	357 ± 16	43,194 ± 2,403	556 ± 9
sCOD	mg/L	185 ± 9	182 ± 6	20,437 ± 1077	273 ± 4
Total nitrogen (TN)	mg/L	52 ± 2	51 ± 6	809 ± 27	58 ± 1
Ammonium-nitrogen (NH_4_-N)	mg/L	38 ± 1	42 ± 1	30 ± 9	40 ± 1
Nitrate (NO_3_^−^)	mg/L	1.53 ± 0.25	1.62 ± 0.06	0.27 ± 0.01	1.59 ± 0.11
Nitrite (NO_2_^−^)	mg/L	< 0.2	< 0.2	< 0.2	< 0.2
Total phosphorus (TP)	mg/L	5.2 ± 0.1	5.1 ± 0.9	114 ± 12	5.8 ± 0.2
Sulfate	mg/L	479 ± 89	436 ± 1	8.75 ± 0.76	418 ± 51
Potassium	mg/L	57 ± 13	76 ± 1	735 ± 6	117 ± 1
Magnesium	mg/L	126 ± 36	156 ± 0.5	54 ± 1	140 ± 24
Calcium	mg/L	116 ± 11	121 ± 4	101 ± 4	116 ± 11
d_50_	nm	2011 ± 206	2276 ± 357	63,727 ± 2750	2851 ± 171
COD/TN	-	6.98 ± 0.22	7.11 ± 0.74	56 ± 6	9.65 ± 0.57

### Laboratory-scale AGS system

**Fig. 2:**
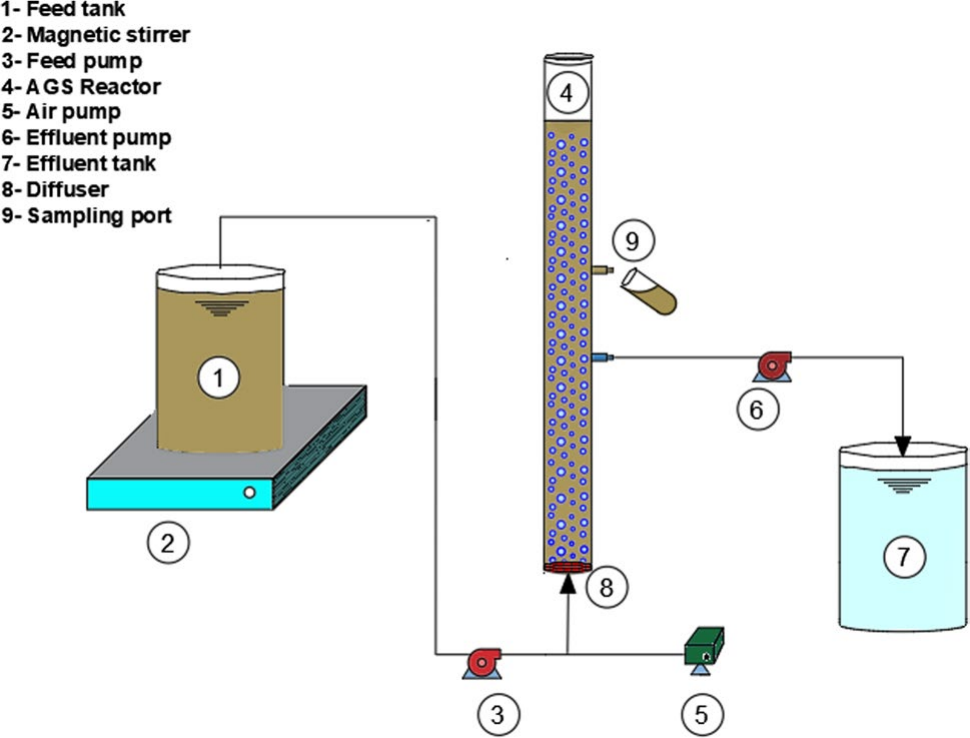
Laboratory-scale AGS system

The laboratory-scale AGS system consisted of a plexiglass reactor with a diameter of 4.5 cm and a water depth of 70 cm. In order to feed wastewater into the reactor from the bottom, a peristaltic feed pump (Watson Marlow SCIQ 323, Belgium) was used (Fig. 2). The discharge was accomplished using a vacuum pump (LongerPump YZ1515x, China). The air was supplied with a fine bubble air diffuser (Atman HP-8000, China) with its header at the bottom of the reactor. The AGS system was kept in a temperature-controlled room between 20 and 25 °C.

### Operational conditions

The AGS reactor was operated with a cycle time of 4 h, consisting of 3 min of feeding, 30 min of settling, 120 min of aeration, 65 min of anaerobic phase, 2 min of idle phase, and 20 min of decanting. The height-to-diameter ratio of the AGS reactor was 16. The AGS system reached steady-state conditions on the 52nd day and then, stable biomass concentration in the reactor and treatment performance were achieved. Afterward, the system was operated for 82 days at stage 1. At stage 2, the FW was mixed with wastewater before feeding to the AGS system. At stage 2, the AGS system was operated for 82 days. COD/TN ratio was monitored during each stage and obtained as 6.98 ± 0.22 at stage 1 and 9.65 ± 0.57 at stage 2. Nitrogen loading rates at stage 1 and stage 2 were 0.14 ± 0.003 kg N/m^3^ day and 0.10 ± 0.002 kg N/m^3^ day, respectively. At stage 1, the phosphorus loading rate was 0.014 ± 0.0004 kg P/m^3^ day, while it was 0.010 ± 0.0003 kg P/m^3^.day at stage 2.

### Experimental analysis

#### Analysis techniques

TSS, VSS, COD, TN, NH_4_-N, and TP parameters were measured based on Standard Methods (APHA 2017). The samples were filtered through 0.45-μm syringe filters before the soluble COD analysis. Concentration of particulate COD (pCOD) was calculated by subtracting soluble COD (sCOD) from total COD concentration. A pH meter (XS pH 50 + DHS, Carpi MO, Italy) was used to determine the pH value. Turbidity was measured by using a turbidimeter (Hach 2100P, USA) having a detection range of 0–1000 NTU. CST analysis was performed using a CST analyzer with an 18 mm funnel (Triton Electronics, Type 304 M, UK). To reduce the impact of suspended solids concentration on CST, the normalized CST value was computed by dividing CST by the TSS concentration in the AGS reactor (Khan et al. 2008; Ersahin et al. 2014). Particle size distribution (PSD) of the AGS was determined with a Mastersizer 2000 (Malvern Instruments, Hydro 2000 MU, UK) with a detection range of 0.6–6000 μm. SVI_30_ measurement was performed based on the Standard Methods (APHA 2017). The values of SVI_5_ and SV_I0_ were measured by reading the height of the settled sludge after 5 and 10 min, respectively (van Loosdrecht et al. 2016). Ion chromatography was used for conducting the measurement of nitrate, nitrite, sulfate, potassium, magnesium, and calcium concentrations (Dionex ICS-300, USA).

The high-temperature sodium carbonate (Na_2_CO_3_) extraction method was used to extract extracellular polymeric substances (EPS) (Felz et al. 2016). Protein (PN) and carbohydrate (PS) fractions of the structural EPS were determined following the methods of Dubois et al. (1956) and the modified Lowry method (Frolund et al. 1995), respectively. The method described by Ghangrekar et al. (1996) was used to determine the integrity coefficient (IC) of the granules. Specific granule density was determined by a pycnometer (van Loosdrecht et al. 2016). Granular sludge was sieved through three different mesh-sized sieves (from top to bottom: 2 mm, 1 mm, and 0.5 mm) (van Loosdrecht et al. 2016). Based on their diameters, granules were categorized into three size classes including 0.5–1 mm, 1–2 mm, and > 2 mm. Granules were randomly chosen from each class and analyzed using light microscopy in order to determine the real diameter (Leica S8APO, Leica Microsystems, Germany).

#### Morphological analyses

The morphology of granules was visualized using an environmental scanning electron microscopy (ESEM) (Thermo Fisher Scientific, FEI Quanta FEG 250, USA). ESEM was combined with an energy-dispersive X-ray (EDX) spectroscopy to identify the major elements on the surface of the granules. Organic materials on the surface of the granules were detected by using Fourier transform infrared spectroscopy (FTIR) (Perkin-Elmer, Spectrum 100, USA).

### BMP test

BMP tests were carried out in triplicate with the Automatic Methane Potential Test System II (AMPTS II) (Bioprocess Control, Sweden). The inoculum was taken from a full-scale anaerobic digester treating municipal wastewater. The ratio of inoculum to the substrate was chosen to be 2:1 on a VS basis. The working volume of the mixture of inoculum and substrates was 400 mL. All bottles were placed in a water bath with a temperature set to 37 °C. Each bottle was sparged with nitrogen gas for 3 min to remove oxygen from the headspace. Phosphate buffer solution, macronutrients, and trace elements were made by following the steps in the study of Zhang et al. (2014). Cellulose microcrystalline (Sigma Aldrich, USA) was used as a model substrate for the positive control (PC) of the BMP tests. The digestion process was simulated using the modified Gompertz model (Filer et al. 2019), as shown in the following equation.

1 $$B\left(t\right)={B}_{0}*exp\left\{-{\text{exp}}\left[\frac{{R}_{m} * {\text{exp}}(1)}{{B}_{0}}*(\lambda -{\text{t}})+1\right]\right\}$$

where *B*(*t*) is the simulated cumulative methane yield (mL CH_4_/g VS), *B*_0_ is the simulated highest cumulative methane yield (mL CH_4_/g VS), *R*_*m*_ is the maximum methane production rate (mL CH_4_/g VS d), *λ* is the lag phase (d), and *t* is the digestion time (d).

### Mass balance

Mass balance was constructed for each configuration given in Fig. 1 to understand the fate of COD. The mass balance was conducted based on the following equation.

2 $${{\text{COD}}}_{{\text{influent}}}={{\text{COD}}}_{{\text{effluent}}}{ +\mathrm{ COD}}_{{\text{sludge}}}{ +\mathrm{ COD}}_{{\text{min}}}$$

where ${{\text{COD}}}_{{\text{influent}}}$ is the influent COD load (g/day), ${{\text{COD}}}_{{\text{effluent}}}$ is the effluent COD load (g/day), ${{\text{COD}}}_{{\text{sludge}}}$ is the COD load in the sludge (g/day), and ${{\text{COD}}}_{{\text{min}}}$ is mineralized COD during oxidation (g/day).

For anaerobic digestion, the COD load in the sludge (${{\text{COD}}}_{{\text{sludge}}}$) (g/day) was considered as the sum of COD which is transformed into methane gas (${{\text{COD}}}_{{\text{methane}}}$) (g/day) and the COD load that remained in the digestate (${{\text{COD}}}_{{\text{digestate}}}$) (g/day). ${{\text{COD}}}_{{\text{methane}}}$ was determined according to the following equation:

3 $${{\text{COD}}}_{{\text{methane}}}={ (Q}_{{\text{methane}}}/0.35) * {{\text{COD}}}_{{\text{sludge}}} * {({\text{VS}}/{\text{COD}})}_{{\text{sludge}}}$$

where ${Q}_{{\text{methane}}}$ (L/g VS) is the amount of methane produced per g VS of the sludge, 0.35 is the theoretical methane generation per g COD at a standard temperature of 273 K and an atmospheric pressure of 1 atm (L methane/g COD), and ${({\text{VS}}/{\text{COD}})}_{{\text{sludge}}}$ is the ratio of VS to COD in the sludge.

## Results and discussions

### Treatment performance

**Fig. 3:**
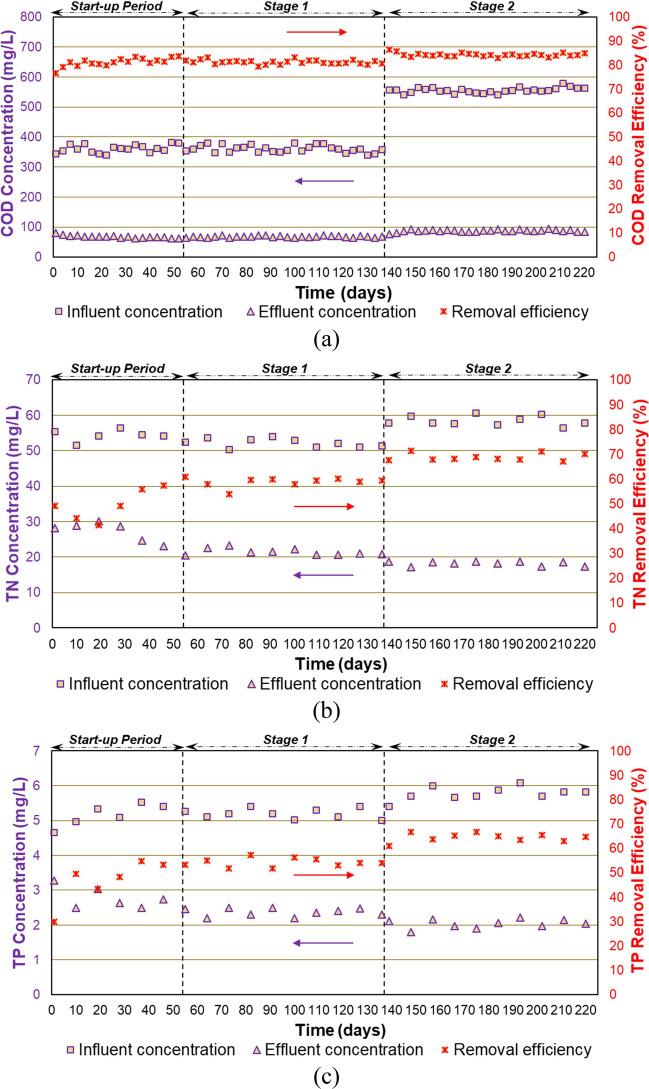
Treatment performance of the AGS system: **a** COD removal, **b** TN removal, and **c** TP removal

Treatment performances achieved at each stage are given in Fig. 3. Addition of FW to wastewater increased the pollutant concentrations in the influent of the AGS system. FW addition increased the average COD concentration in influent from 361 to 556 mg/L. The increase in pCOD concentration would be more than the increase in sCOD concentration after FW addition due to the higher pCOD/COD ratio in pulped FW (Guven et al. 2019). pCOD and TSS concentrations in the influent increased by 61% and 42% at stage 2, respectively. The increase in pCOD concentration was significantly influenced by the amount and composition of FW (Table 2). Marashlian and El-Fadel (2005) and Monino et al. (2017) found that the TSS concentration of the FW ranges between 1537 and 39,480 mg/L, which was 19,570 ± 625 mg/L in this study (Table 2). COD removal efficiencies were over 80% at each stage under steady-state conditions (Fig. 3a). The removal efficiency of COD was 85 ± 1% at stage 2, despite the contribution of FW in the organic load. A long anaerobic phase is necessary in the AGS process especially for the treatment of wastewaters including particulate matter in order to ensure enough time for the hydrolysis of particulates to soluble forms, which is available for anaerobic microorganisms inside the granules (Wagner et al. 2015). By minimizing the attached particulate matter on the granule surface during the aerobic phase, it is possible to prevent the growth of ordinary heterotrophic microorganisms promoting degranulation and growth of filamentous. The anaerobic period (65 min) in this study was enough to hydrolyze particulate matter at stage 2.

Average TN removal efficiency was obtained to be 63 ± 2% and 75 ± 2% at stage 1 and stage 2, respectively (Fig. 3b). It was enough slowly growing nitrifiers for completing nitrification at each stage because the NH_4_-N removal was nearly complete (> 99%). Ni et al. (2009) reported that the AGS process treating domestic wastewater had ~ 95% of NH_4_-N removal efficiency, which is consistent with the results obtained in this study. Nitrite concentrations were less than 0.05 mg NO_2_-N/L at each stage in the influent and effluent. Nitrite concentration in the effluent of an AGS process fed with synthetic municipal wastewater was found less than 0.06 mg NO_2_-N/L (Kosar et al. 2022b). Nitrate concentration in the effluent was 15.4 ± 1.1 mg NO_3_-N/L at Stage 1, whereas it was 9.9 ± 0.8 mg NO_3_-N/L at Stage 2. The reason for the lower removal efficiency of TN at stage 1 was associated with inadequate denitrification, which might be confirmed by higher nitrate concentrations in the effluent at stage 1. It was observed that the majority of the TN corresponded to NO_2_-N and NO_3_-N, indicating incomplete denitrification (Barrios-Hernández et al. 2020). Pulped FW was not rich in nitrogen and phosphorus. Thus, FW addition to wastewater had little impact on TN and TP concentrations in the influent. Influent TN and TP concentrations at stage 2 were similar with those at stage 1; therefore, the COD/TN ratio of the influent at stage 2 increased from 6.98 ± 0.22 to 9.65 ± 0.57. An increase in the COD/TN ratio provided a better denitrification efficiency and lower NO_3_-N concentration in effluent at stage 2 compared to stage 1. The higher TP and TN removal efficiencies obtained at stage 2 might be due to sufficient carbon availability for PAOs and anoxic denitrifiers. The increase in the COD/TN ratio led to an enhancement in the removal efficiency of TN from 63 ± 2% to 75 ± 2% at stage 2. Kim et al. (2021) reported that TN removal efficiency increased from 57.5 to 79.1% with an increase in the COD/TN ratio from 5 to 20. TP concentrations were 2.07 ± 0.11 mg/L and 1.65 ± 0.09 mg/L in the effluent and TP removal efficiencies were 60 ± 2% and 71 ± 2% at stage 1 and stage 2, respectively (Fig. 3c). Effluent TP concentrations were reported in the range of 0.9–3.2 mg/L in full-scale AGS processes treating domestic wastewater (Giesen and Thompson 2013; Pronk et al. 2017).

**Fig. 4:**
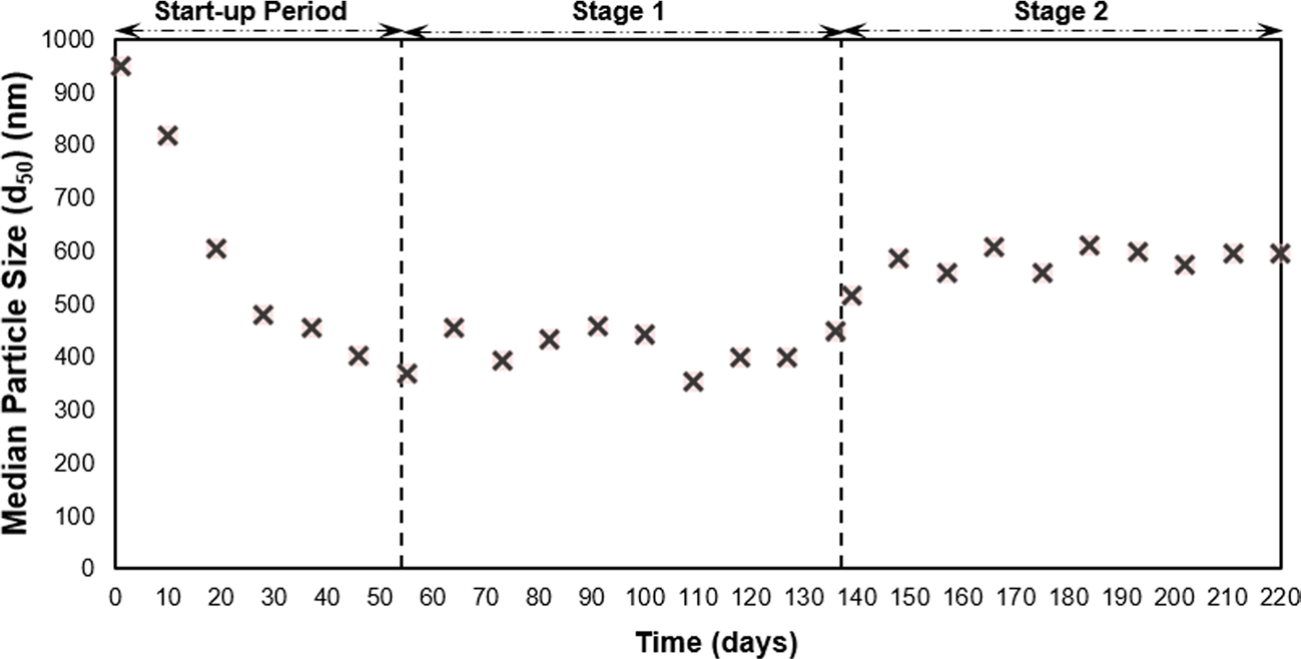
Median particle size (d_50_) in the effluent of AGS system

After steady-state conditions, the average TSS concentration in the effluent was 17 ± 2 mg/L at Stage 1, while it was 33 ± 4 mg/L at stage 2. The addition of FW to wastewater might be the reason behind the higher effluent TSS concentration obtained at stage 2. Similar average removal efficiencies of TSS were achieved at each stage (92.9 ± 1.1% and 90.4 ± 1.2% at stage 1 and stage 2, respectively). The average turbidity in the influent was 209 ± 9 NTU, while it was 3.5 ± 0.1 NTU in the effluent at Stage 1. At stage 2, the turbidity of the influent was 236 ± 9 NTU, and it reduced to 5.4 ± 0.3 NTU in the effluent. Based on particle size distribution, d_50_ in the influent was 2011 ± 206 nm at Stage 1, while it was 2851 ± 268 nm at stage 2. d_50_ in the effluent decreased to 414 ± 35 nm and 581 ± 27 nm at the end of the operation at stage 1 and stage 2, respectively (Fig. 4).

### Granule formation and structure

#### Physical and chemical characteristics of granules

**Fig. 5:**
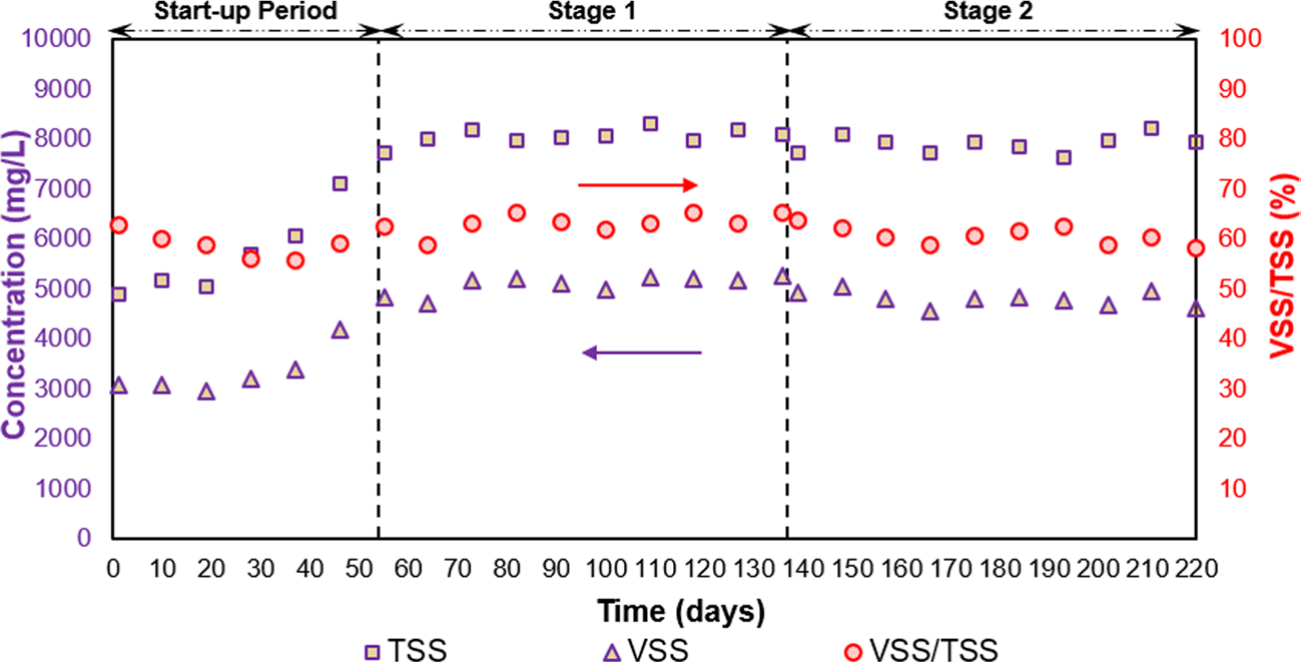
Biomass concentration in the reactor

Average TSS concentrations in the AGS process were similar during feeding with only raw wastewater, and the mixture of the raw wastewater and FW (8077 ± 159 mg/L at stage 1 and 7916 ± 171 mg/L at stage 2) (Fig. 5). VSS/TSS ratio was 63 ± 3% at stage 1 and 61 ± 2% at stage 2. The SVI parameter, which is measured after 5 min, 10 min, and 30 min of the settling (SVI_5_, SVI_10_, and SVI_30_, in order), is an indicator of sludge settleability. The ratio of SVI_30_/SVI_5_ and SVI_30_/ SVI_10_ was close to 1 in this study, indicating a fast settling of well-granulated sludge property (Ekholm et al. 2022). Excess sludge from the AGS process exhibited excellent settleability with SVI_30_ value of 54 ± 3 mL/g at stage 1, and 48 ± 3 mL/g at stage 2. At each stage, SVI_30_ values for the AGS process consistent were with the literature (30–67 mL/g) (Bengtsson et al. 2018). The ratio of SVI_30_/SVI_5_ was calculated to be 0.81 ± 0.04 and 0.87 ± 0.06 at stage 1 and stage 2, respectively (Table 3). Normalized CST was 1.03 ± 0.04 s/g TSS/L at stage 1 and 0.96 ± 0.06 s/g TSS/L for stage 2. Following the normalized CST, it could be inferred that the dewaterability and filterability characteristics of the excess sludge from the AGS process at stage 2 were better than those at stage 1. Higher d_50_ values of the excess sludge were obtained at stage 2 (92 ± 11 μm) compared to stage 1 (80 ± 7 μm) (Table 3; Fig. S1). A key factor for the denotation of nitrogen removal efficiency is the size of an aerobic granule (Bathe et al. 2005). Small granules allow oxygen to penetrate inner layers, while big granules can include anoxic/anaerobic zones to complete denitrification (Nguyen Quoc et al. 2021). An increase in the ratio of COD/TN in the influent from 6.98 (stage 1) to 9.65 (stage 2) led to an increase in both granule size and capacity of denitrification. A similar finding was found by Kosar et al. (2023) that the ratio of nitrifiers to heterotrophs could rise and, denitrifiers could be dominated by typical aerobic heterotrophs due to the lack of available carbon in the influent and hence, the growth of granules might be hampered by a lack of adequate anoxic/anaerobic volume in the granule core. Sufficient carbon can be provided for denitrifiers by increasing the COD/TN ratio (Kosar et al. 2023).

**Table 3:** Characteristics of the excess sludge from the AGS process

Parameter	Unit	Stage 1 (average ± SD)	Stage 2 (average ± SD)
COD	mg/L	8286 ± 505	8237 ± 332
SVI_5_	mL/g	81 ± 8	68 ± 7
SVI_10_	mL/g	68 ± 4	56 ± 5
SVI_30_	mL/g	54 ± 3	48 ± 3
SVI_30_/SVI_10_	-	0.68 ± 0.07	0.71 ± 0.07
SVI_30_/SVI_5_	-	0.81 ± 0.04	0.87 ± 0.06
Normalized CST	s/g TSS/L	1.03 ± 0.04	0.96 ± 0.06
pH	-	7.3 ± 0.2	6.9 ± 0.2
d_50_	µm	80 ± 7	92 ± 11

**Fig. 6:**
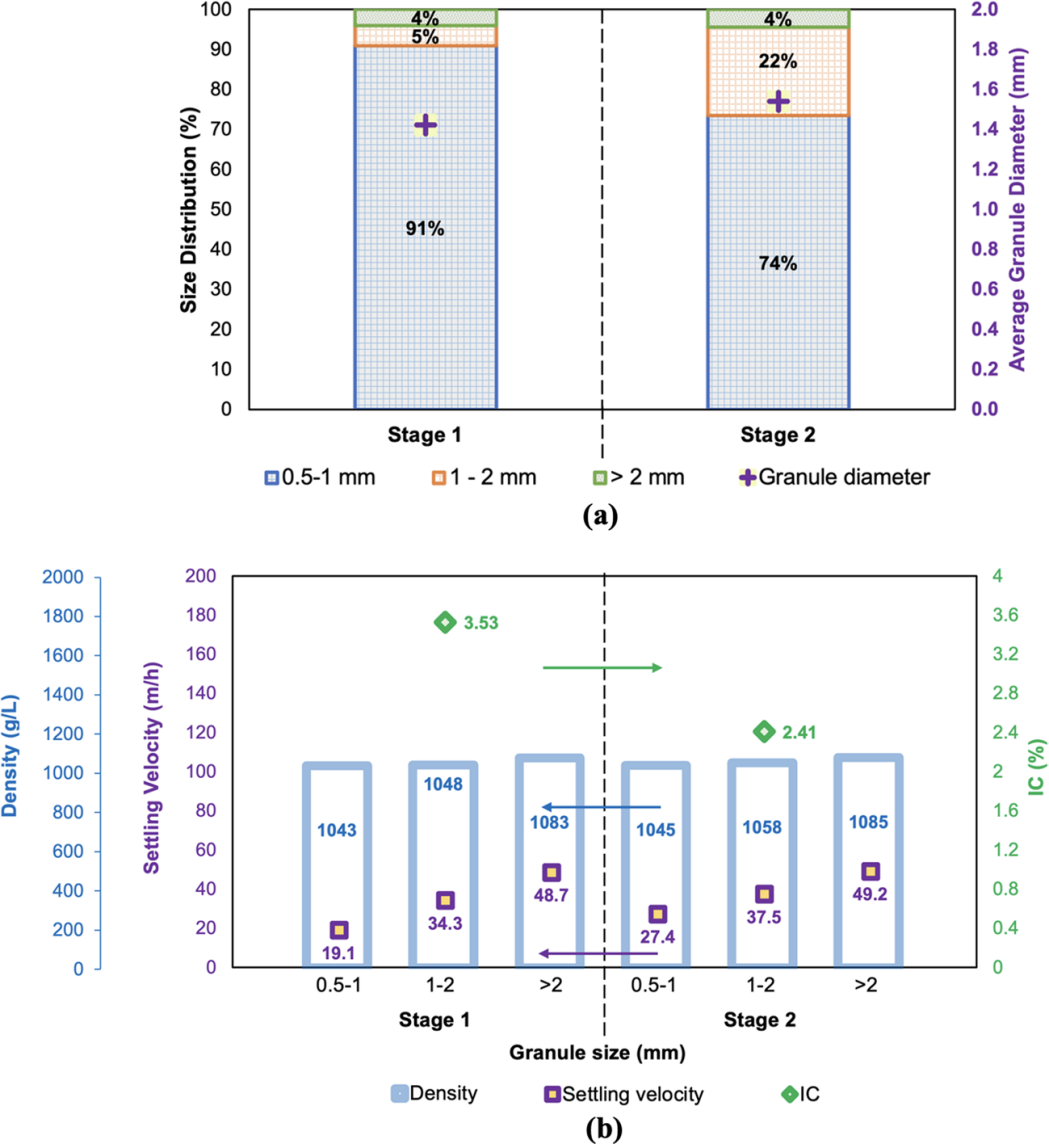
Properties of the granules: **a** size distribution; **b** physical characteristics

Aerobic granules typically range in diameter from 0.2 to 3 mm. (Bengtsson et al. 2018). Zhou et al. (2016) found that the optimum size of the granular range is 0.7–1.9 mm to enhance nitrogen removal efficiency. At stage 1, a mean granule diameter of 1.42 mm was obtained. The granules (91%) were in a range of 0.5–1.0 mm. However, the granule size had more variation at stage 2. Mean granule diameter of 1.54 mm was obtained at stage 2, and only 74% of granules were in a range of 0.5–1 mm. The granules (22%) were in a range of 1–2 mm, and the remaining granules had diameter > 2 mm at stage 2 (Fig. 6a). Better TN removal efficiency achieved at stage 2, compared to stage 1, might be associated with that more granules were in the range of 1–2 mm at stage 2. The physical characteristics of the aerobic granules are given in Fig. 6b. Granule densities ranged between 1043 and 1069 kg/m^3^ at both stages in this study. This finding was in agreement with the results of van den Berg et al. (2022) in which granule densities were in the range of 1005–1070 kg/m^3^. Settling velocities of 19.1–27.4 m/h, 34.3–37.5 m/h, and 48.7–49.2 m/h were found for granules in the size range of 0.5–1 mm, 1–2 mm, and > 2 mm, respectively. Feng et al. (2021) found that settling velocities ranged between 25 and 70 m/h. In this study, the IC of the aerobic granules at stage 1 and stage 2 was found 3.53 ± 0.39% and 2.41 ± 0.23%, respectively. Granules having low IC values represent higher strength compared to those that have higher IC values (Ghangrekar et al. 1996).

### Morphology of granules

**Fig. 7:**
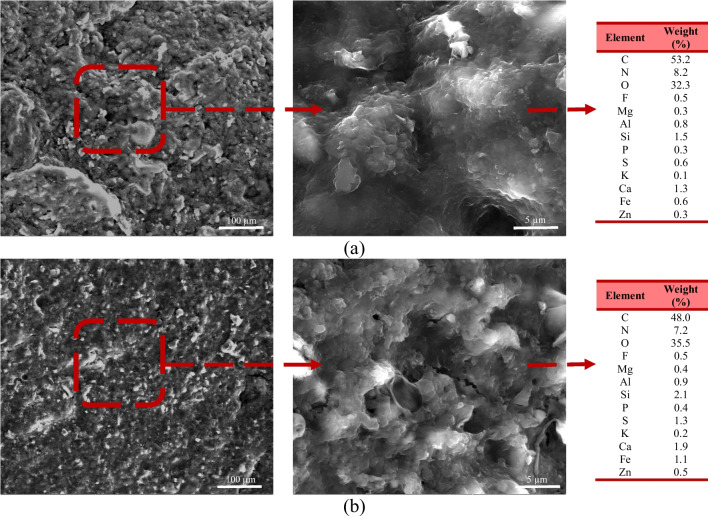
ESEM images of the granules and EDX results: **a** Stage 1; **b** Stage 2

The surface of the granules was imaged by ESEM/EDX at the end of each stage (Fig. 7; Fig. S2). Surface of the granules was remarkably different at stage 2 compared to stage 1. The structure of granules obtained at stage 2 was smoother than those at stage 1. EDX analysis was utilized to describe the elemental composition of the granules. Based on the EDX results, elements such as O, C, and N were the main components in the granules at each stage, forming more than 90% of the elements on the granule surface. A relatively high percentage of C (stage 1: 53.2%; stage 2: 48.0%) and N (stage 1: 8.2%; stage 2: 7.2%) could be associated with the high organic content of the influent. The availability of minerals promotes the aggregation of microorganisms, which contributes to the aerobic granulation process. Divalent and trivalent metal ions act as a bridging mechanism between negatively charged ions on the cell surface which positively impact the aggregation of the granules (Li et al. 2014). Mg and Ca have a considerable impact on augmenting the granulation process (Jiang et al. 2003; Ren et al. 2008). Moreover, Al and Fe are essential in developing more compact and settleable granules (Li et al. 2014). Othman et al. 2013 showed that aerobic granule structure was positively affected by Si, which also supported the strength of the matured granules.

**Fig. 8:**
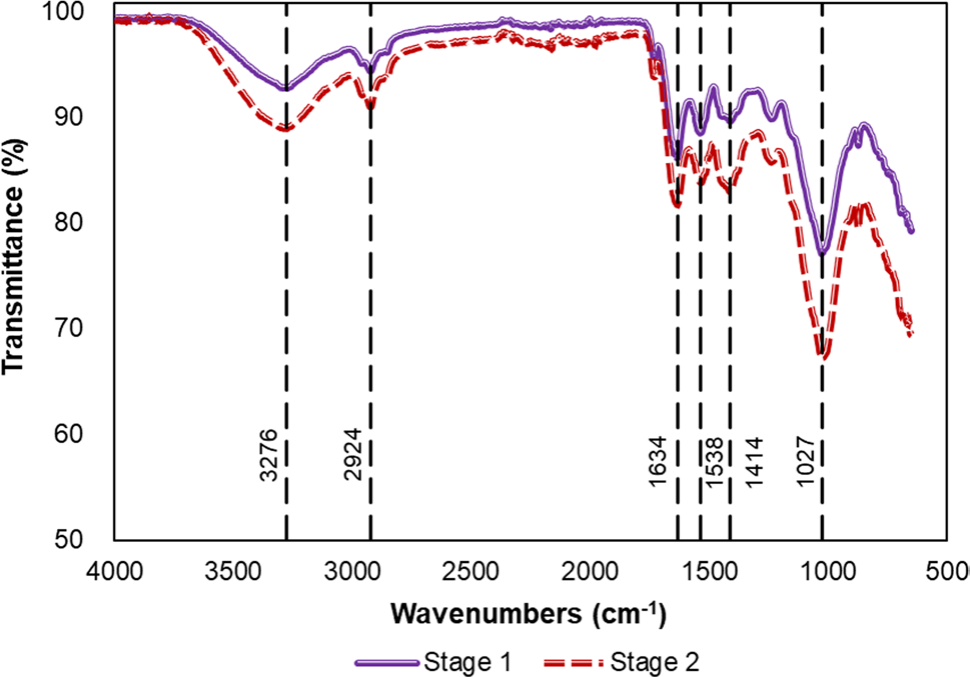
FTIR spectrum of the granules

Similar peaks on the FTIR spectra were observed at each stage, which suggested that the surface of aerobic granules had similar functional groups (Fig. 8). The peaks at 3276 cm^−1^ in the spectrum showed stretching of the O–H stretching of the amide groups, which indicated the presence polysaccharide in the samples (Liu et al. 2015). The peak at 2924 cm^−1^ was associated with aliphatic C-H stretching (Isik et al. 2019). The structure of the protein was illustrated by the presence of amide groups. Amides I (stretching of C = O and C–N bonds) corresponded to the peak at 1634 cm^−1^. Amides II (deformation of N–H and C = N bonds) corresponded to peaks at 1538 cm^−1^ (Isik et al. 2020). The peaks at 1416 cm^−1^ represented Amides III (C–N stretching) (Abdelrahman et al. 2022). The peak 1027 cm^−1^ indicated the presence of C–OH stretching, representing polysaccharides (Allen et al. 2004; Low et al. 2021). These results of FTIR analysis illustrated the presence of polysaccharides-like and protein-like substances in the aerobic granules, which was expected since EPS likely accumulates on the surface of the aerobic granules.

**Fig. 9:**
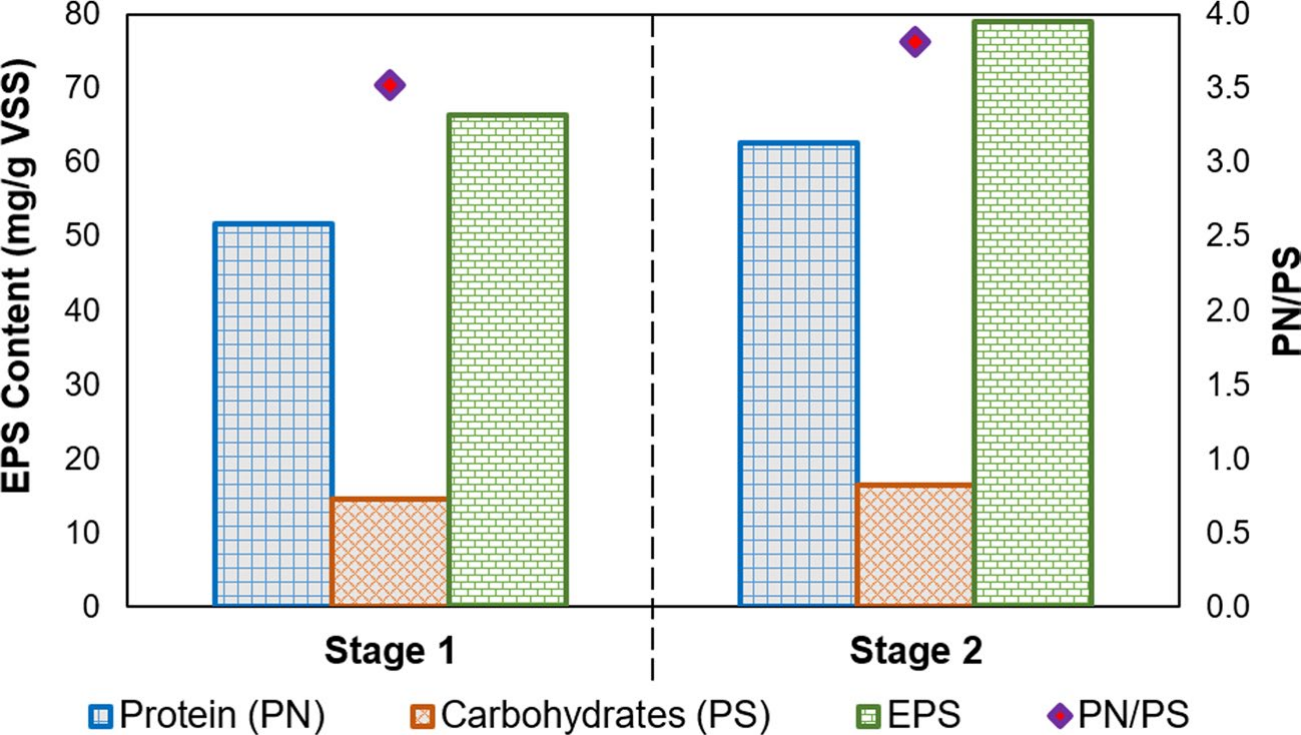
EPS content and PN/PS ratio

EPS, which are gel-like materials secreted either by cells or formed as a result of cell lysis, plays a key function in the growth of microorganisms. Thus, the interaction between EPS and microbial cells has an important impact on the formation of excess sludge from the AGS process (Flemming et al. 2007; Chen et al. 2010; Ni 2013). The results of FTIR highlighted EPS presence at each stage. PN and PS contents are indicated in Fig. 9. PN increased from 51.7 ± 0.8 mg/g VSS to 62.6 ± 1.9 mg/g VSS, while PS slightly increased from 14.7 ± 0.5 mg/g VSS to 16.4 ± 0.3 mg/g VSS at stage 2. Accordingly, the PN/PS ratio was 3.5 ± 0.1 and 3.8 ± 0.2 at stage 1 and stage 2, respectively. The increased EPS amount might be mainly made up of the increase in the production of PN at stage 2. A higher PN/PS ratio provides better granulation (Zhang et al. 2017). Relatively stable PS content at each stage indicated that FW addition did not substantially alter the PS content in the aerobic granule. This finding is consistent with the study of Liang et al. (2021), in which FW addition contributed to the PN content rather than PS content. Consequently, FW addition to an AGS system might increase the protein-based EPS content.

### Biochemical methane potential

**Fig. 10:**
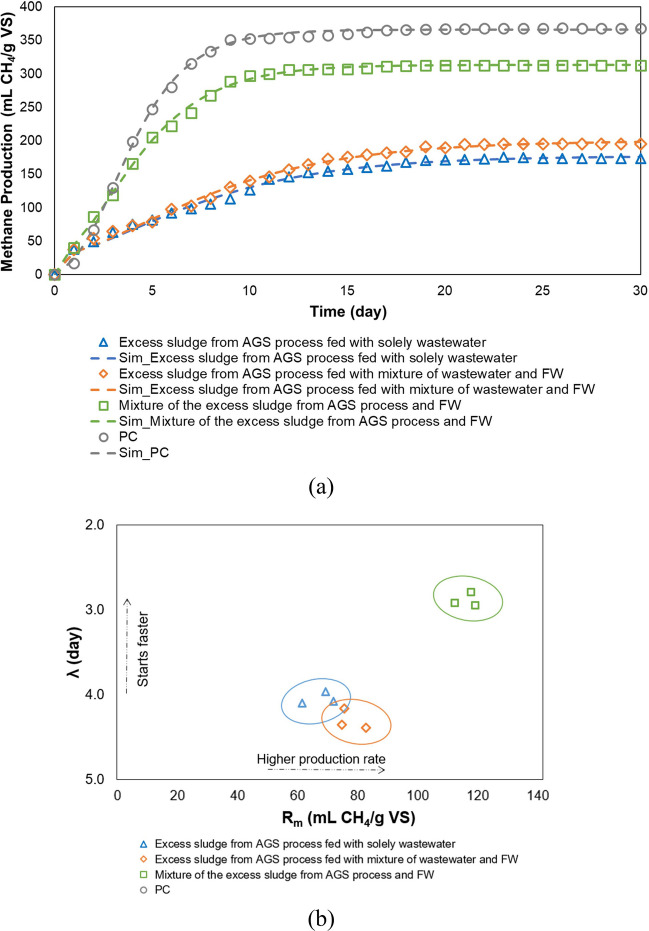
BMP results: **a** experimental and simulated results; **b** correlation between *R*_*m*_ and *λ* (three markers indicate triplicate samples)

Digestibility of the excess sludge from the AGS process was evaluated via the BMP test (Fig. 10). The BMP of the PC was 368 ± 13 mL CH_4_/g VS, on average. It was reported by Holliger et al. (2021) that the BMP of PC should be in the range of 340 and 395 mL CH_4_/g VS, which was fulfilled in this study. The highest BMP value was obtained with the mixture of the excess sludge from the AGS process and FW, which was 312 ± 8 mL CH_4_/g VS. Co-digestion of FW provides higher methane yield and accelerates methane production rate because of a synergistic effect that balances in nutrients and improves biodegradation (Prabhu and Mutnuri 2016). Co-digestion of FW with sewage sludge could enhance methane production by 35–48% (Fitamo et al. 2016). A higher BMP value was obtained from the co-digestion of FW with the excess sludge from the AGS process than the other scenarios. This result could be linked with the high COD/TN ratio in FW, resulting in an enhancement of nutrient balance. BMP of the excess sludge from the AGS process fed with the mixture of wastewater and FW (195 ± 17 mL CH_4_/g VS) was slightly higher than BMP of excess sludge from the AGS process fed with solely wastewater (173 ± 16 mL CH_4_/g VS) (Fig. 10a). Guo et al. (2020a) found that the BMP value for the excess sludge from an AGS process was 232 ± 11 CH_4_/g VS. Higher BMP value observed in the study of Guo et al. (2020a) compared to our study might be related with the higher COD concentration in the excess sludge (71.3 ± 0.4 g/L), which was around 8 g/L in our study (Table 2).

The modified Gompertz model was used to determine methane production since different methane production rates and lag phases were observed. The methane production curves were well-fitted with the modified Gompertz model (*R*^2^ > 0.98 for all curves) (Fig. 10a). The excess sludge from the AGS process fed with solely wastewater had the lowest Rm (4.2 ± 0.5 mL CH_4_/g VS day) and the highest *λ* (4.0 ± 0.1 day). The kinetics of the excess sludge from the AGS process fed with the mixture of wastewater and FW had Rm and *λ* of 5.4 ± 0.3 mL CH_4_/g VS day and 4.3 ± 0.1 days, respectively. The shortest lag phase was observed during the mixture of the excess sludge from the AGS process and FW digestion (2.9 ± 0.1 day) (Fig. 10b).

### Mass balance

**Fig. 11:**
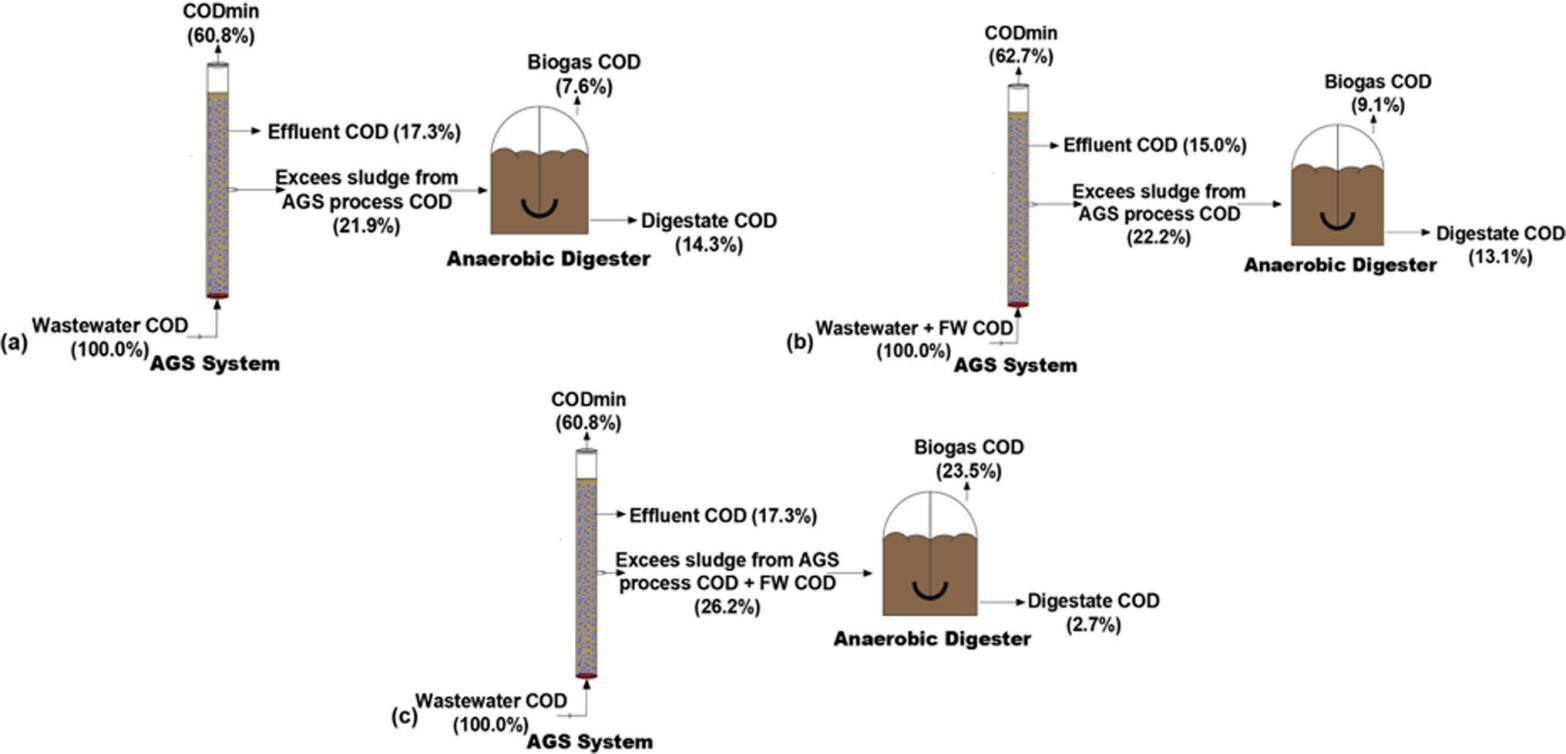
COD mass balance: **a** Excess sludge from the AGS process fed with solely wastewater; **b** excess sludge from the AGS process fed with the mixture of wastewater and FW; **c** excess sludge from the AGS process and FW mixture

A mass balance for the COD parameter was set up to have a better understanding of the fate of the carbonaceous organic matter (Fig. 11). AGS system fed with solely wastewater had a COD removal efficiency (83%), whereas the COD removal efficiency of the AGS system fed with the mixture of wastewater and FW was 85%. AGS system fed with solely wastewater redirected less COD to sludge for anaerobic digestion since 60.8% of COD was used during the metabolic activities which were converted to CO_2_. Similarly, it was 62.7% for the AGS system fed with the mixture of wastewater and FW. COD (44–61%) is transformed into CO_2_ for the AGS process in the literature (Jahn et al. 2019; Guo et al. 2020b). Based on the COD mass balance, only 7.6% of COD could be converted into methane gas if the excess sludge from the AGS system fed with solely wastewater was digested. The excess sludge from the AGS system fed with the mixture of wastewater and FW could recover 9.1% COD from wastewater, for subsequent conversion into methane gas. Moreover, 23.5% of COD could be converted into methane gas if the excess sludge from the AGS process and FW are co-digested.

## Conclusions

In this study, the impact of co-treatment of wastewater and FW on the treatment performance, and granule morphology was assessed, and a BMP assay was conducted to determine the methane potential of mono- and co-digestion of the excess sludge from the AGS process. Better nutrient removal efficiencies and granule strength were achieved in the AGS process co-treating of the wastewater and FW (stage 2) compared to the AGS process treating solely wastewater (stage 1). The highest methane yield was obtained for excess sludge from the AGS system and FW mixture with the value of 312 ± 8 mL CH_4_/g VS. Both the addition of FW to wastewater and the mixture of the excess sludge from the AGS process and FW could enhance energy content by adding more biodegradable matter, which can increase energy recovery potential in anaerobic digestion. Hence, co-digestion of FW with the excess sludge from the AGS process can be a beneficial approach for maximizing energy recovery for the treatment of municipal wastewater.

### Supplementary Information

Below is the link to the electronic supplementary material.

Supplementary file1 (DOCX 8774 KB)

## Data Availability

No supplementary information.
