# Supplementary material for: Impact of food waste addition in energy efficient municipal wastewater treatment by aerobic granular sludge process
Source: Environ Sci Pollut Res Int. 2024 Apr 4;31(20):29304–20. doi: 10.1007/s11356-024-32997-5 (PMC11058935; doi:10.1007/s11356-024-32997-5)
Supplement: Supplementary file 1 — Supplementary file1 (DOCX 8774 KB) [file 11356_2024_32997_MOESM1_ESM.docx]

**Impact of food waste addition in energy efficient municipal wastewater treatment by aerobic granular sludge process**

Busra Cicekalan^1,*^, Nastaran Rahimzadeh Berenji^1^, Muhammed Furkan Aras^1^, Huseyin Guven^1^, Ismail Koyuncu^1,2^, Mustafa Evren Ersahin^1,2^, Hale Ozgun^1,2^

^1^Istanbul Technical University, Civil Engineering Faculty, Department of Environmental Engineering, Maslak, 34469, Istanbul, Turkey

^2^National Research Center on Membrane Technologies, Istanbul Technical University, Maslak, 34469, Istanbul, Turkey

* Corresponding author: cicekalan@itu.edu.tr


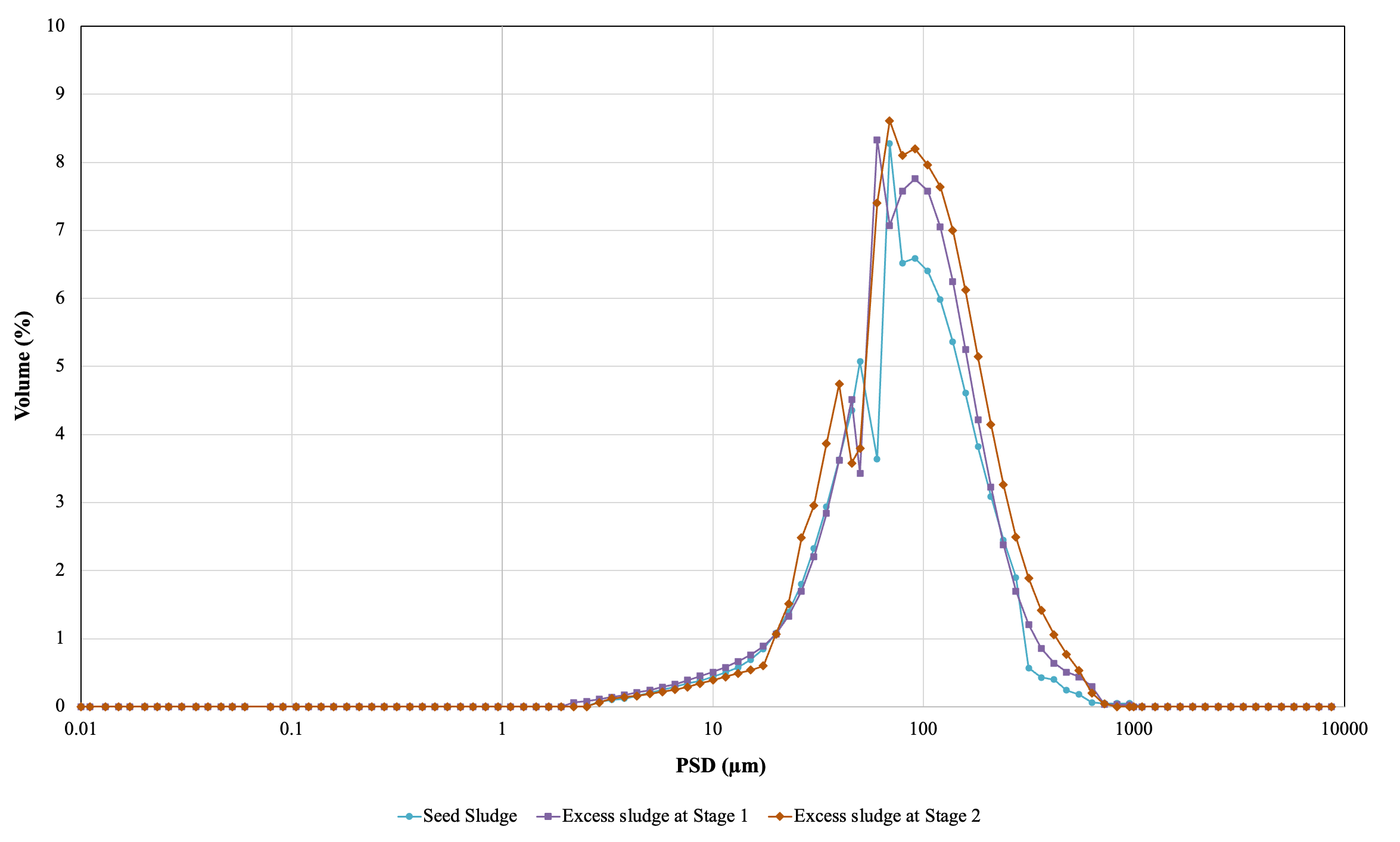


**Fig. S1.** Particle size distribution of excess sludge from AGS process

| **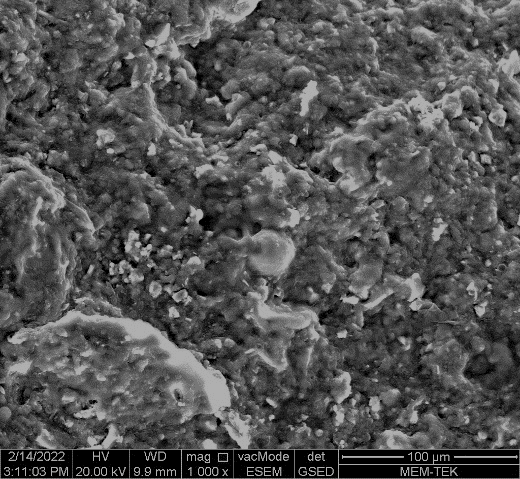**  100 µm | **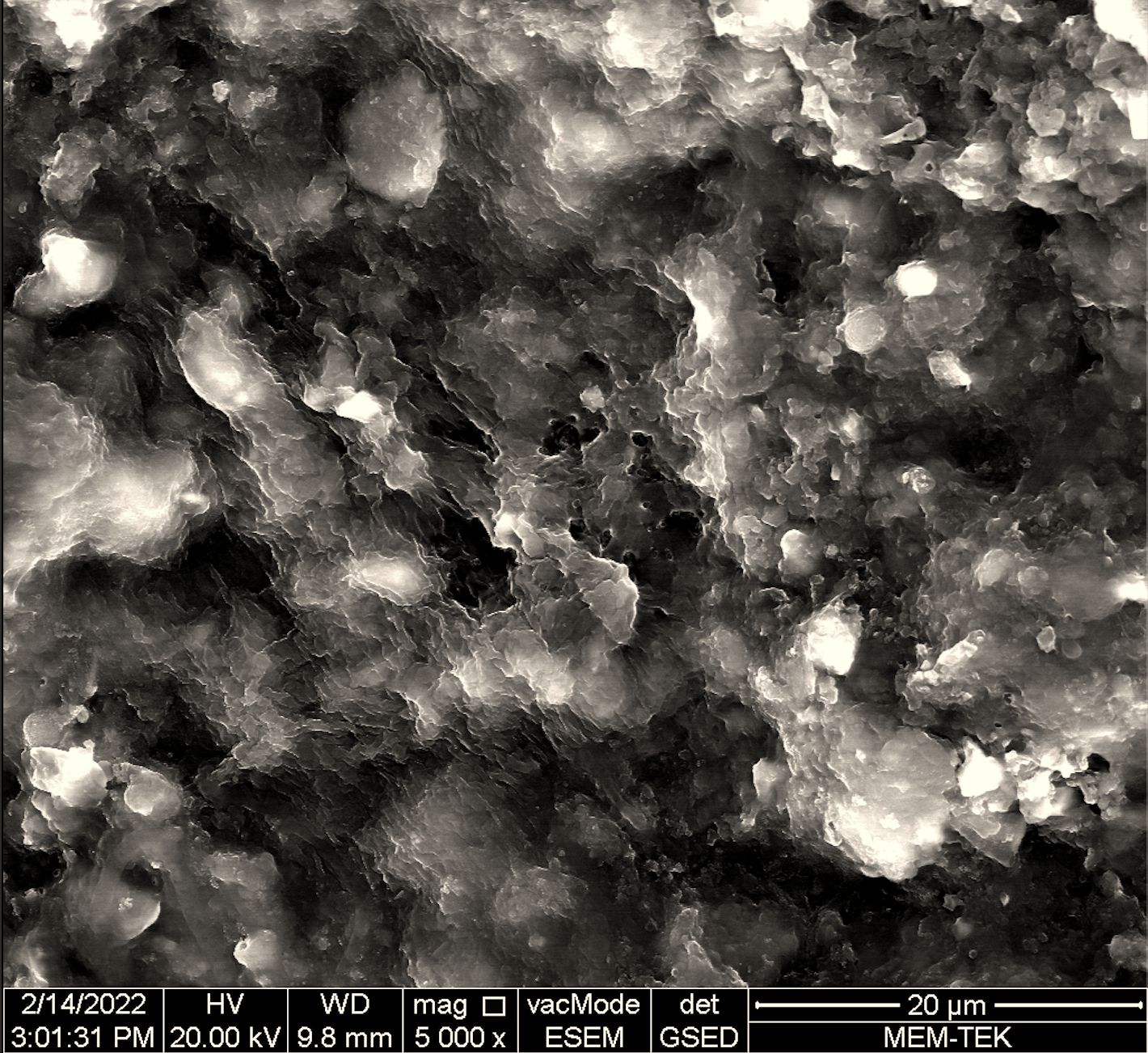**  20 µm | **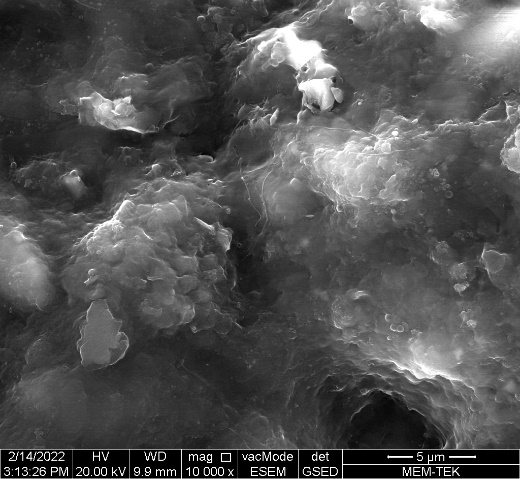**  5 µm | **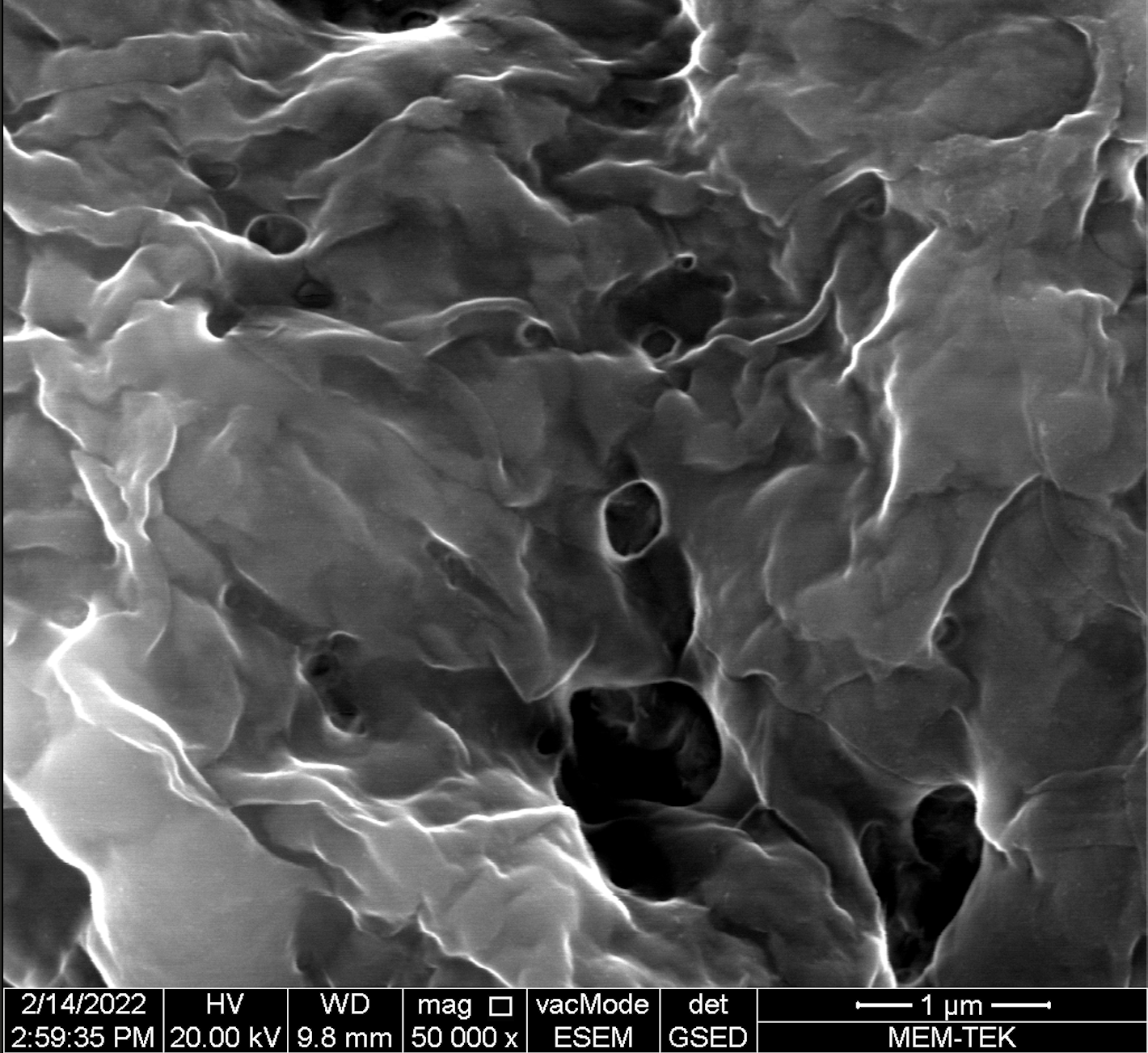**  1 µm |
| --- | --- | --- | --- |
| (a) | | | |
| **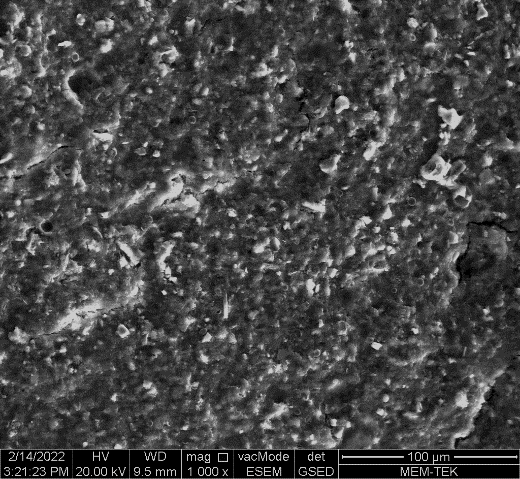**  100 µm | **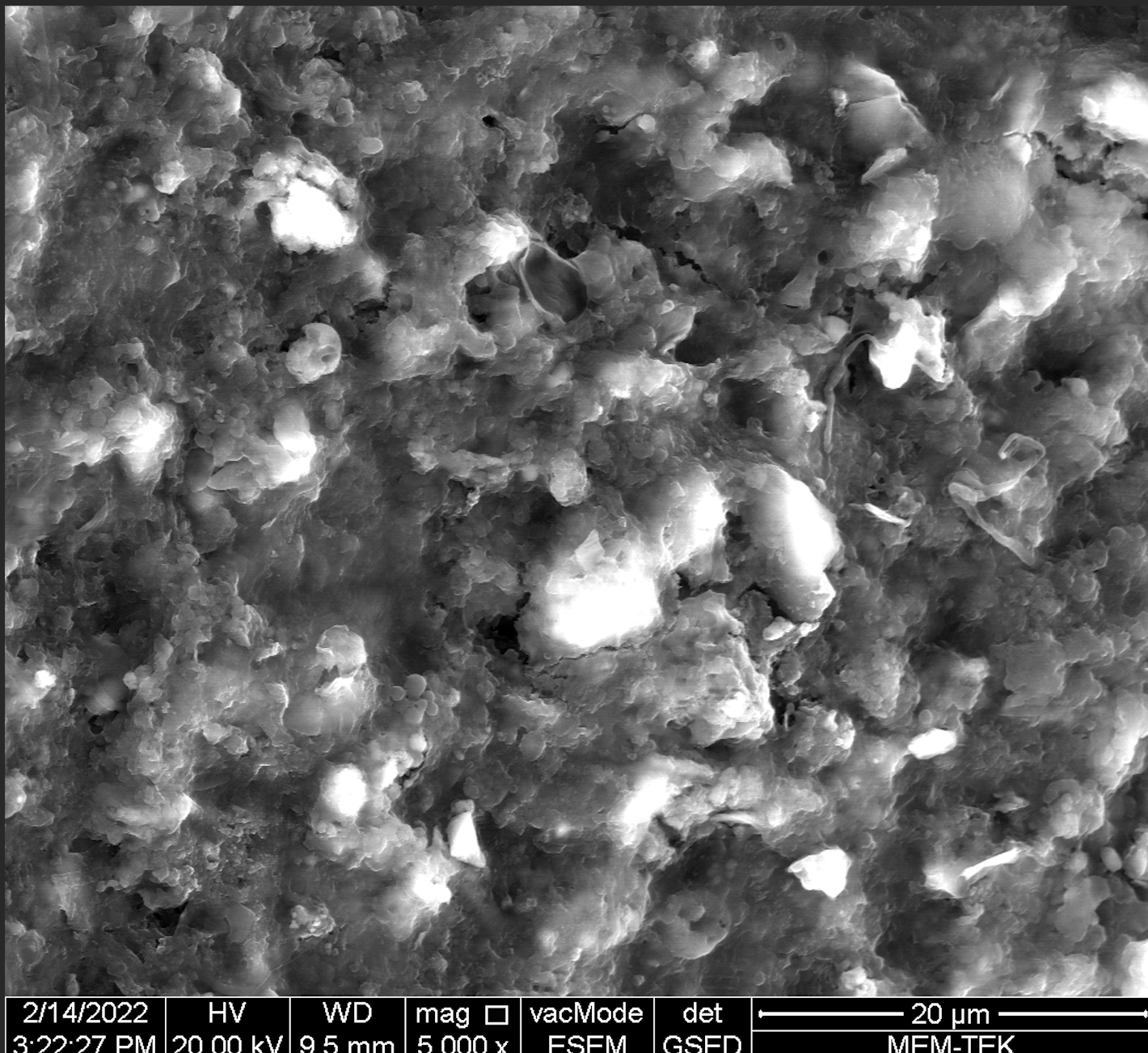**  20 µm | **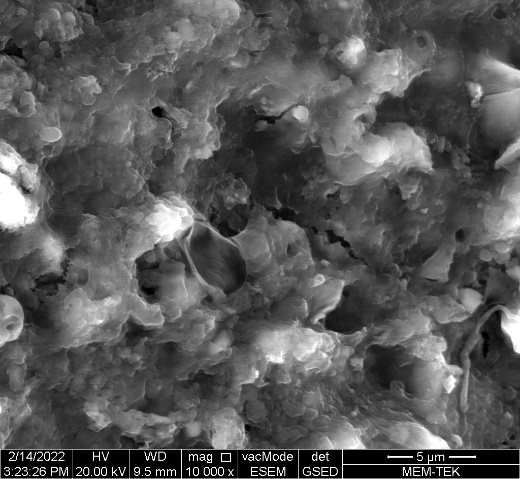**  5 µm | **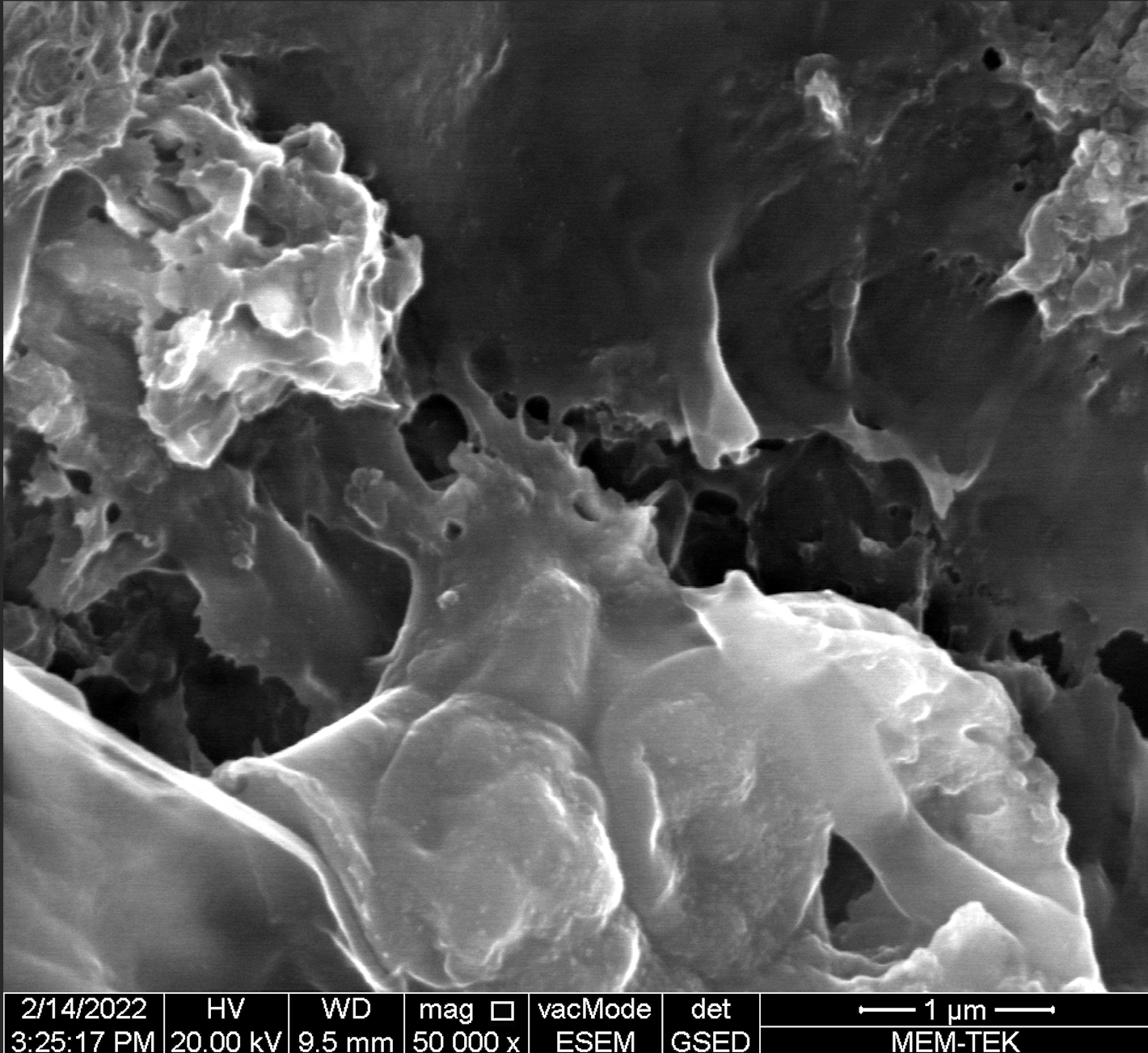**  1 µm |
| (b) | | | |

**Fig. S2.** ESEM images of the granules under different magnifications: (a) Stage 1, (b) Stage 2.
